# Association of HCV Infection with C-Reactive Protein: National Health and Nutrition Examination Survey (NHANES), 2009–2010

**DOI:** 10.3390/diseases7010025

**Published:** 2019-02-24

**Authors:** Azad R. Bhuiyan, Amal K. Mitra, Oluwabunmi Ogungbe, Nusrat Kabir

**Affiliations:** Department of Epidemiology and Biostatistics, School of Public Health, Jackson State University, Jackson, MS 39213, USA; amal.k.mitra@jsums.edu (A.K.M.); oluwabunmi.v.ogungbe@students.jsums.edu (O.O.); nusrat.kabir@students.jsums.edu (N.K.)

**Keywords:** hepatitis C virus, C-reactive protein, HCV-RNA, inflammation, NHANES data

## Abstract

The relationship between hepatitis C virus (HCV) infection and C-reactive protein (CRP), which is an inflammatory biomarker, is limited in studies with the general population. It was hypothesized that changes in CRP levels are genotype-dependent in the general population with HCV infection. Thus, this study aimed to assess the prevalence of HCV infection and compare CRP levels with an anti-HCV antibody, HCV-RNA status, and HCV genotypes. A total of 5611 adult participants from the National and Health Nutrition Examination (NHANES), 2009–2010 survey were analyzed. Proc survey frequency, means, and multivariate regression were used due to the complex survey design of NHANES. The prevalence of HCV infection among the study population was 1.6%. There were lower mean CRP levels among people with anti-HCV antibody positive status compared to those with antibody negative status (0.12 ± 0.08 vs. 0.24 ± 0.02, *p* = 0.08, 95% Confidence Intervals, CI: −1.12 to 0.07). Mean CRP levels were also lower in people with HCV-RNA positive status compared to those with HCV-RNA negative status (0.56 ± 0.03 vs. 0.48 ± 0.05, *p* = 0.62 and 95% CI: −1.37 to 0.86). However, these differences were non-significant. With respect to HCV genotypes, significantly higher CRP levels were noted among people infected with HCV genotype 2 vs. genotype 1 (0.53 ± 0.06 vs. 0.23 ± 0.05, *p* < 0.01, 95% CI: −0.58 to −0.02) and those with HCV genotype 2 vs. HCV genotype 3 (0.53 ± 0.06, 0.28 ± 0.04, *p* < 0.01, 95% CI: 0.02 to 0.48). Further studies are needed to confirm this finding.

## 1. Introduction

Hepatitis C virus (HCV) infection is the most common blood-borne infection in the United States and worldwide [1]. Globally, seventy-one million people are infected with chronic infections due to HCV [2]. More than 3.5 million people in the USA are suffering from HCV, with 17,000 new cases occurring each year, and many are underreported [3]. Among people infected with HCV, 80% develop chronic infection, 20% develop cirrhosis, 10% go on to develop end-stage liver disease or cancer, and 3–4% are recommended for a liver transplant or eventually die from complications related to the infection [4]. From 2011–2014, there was a 250% increase in new HCV infections [5] and deaths have increased by 20% from 2010 to 2014 because most of the infected individuals were not aware of their HCV infection state, and neither did they seek treatment [6].

According to the Centers for Disease Control and Prevention (CDC) and World Health Organization (WHO) reports, the most common modes of HCV infection are hazardous lifestyle behaviors such as injection drug use, sharing syringes and needles and high-risk sexual behaviors. The infection is also prevalent among the younger generation and baby boomers born between 1945–1965, the recipients of donated blood or organs from a person who is infected with HCV and people working in the health field who are at risk of needle pricks, as well as people who receive body piercings or tattooing with non-sterile tools [2,3,6,7].

Chronic inflammation plays a vital role in the pathogenesis of atherosclerosis [8,9,10]. Biomarkers such as oxidized low-density lipoproteins, interleukin-6, intercellular adhesion molecule-1 and high sensitivity C-reactive protein (CRP) can reflect ongoing inflammatory process [9,11]. The CRP—an acute-phase reactant secreted by the liver—has emerged as an independent predictor of cardiovascular disease, type 2 diabetes, and chronic kidney disease [9,10,12,13,14,15,16]. HCV infection is also a risk factor for atherosclerosis [17]. Consequently, the American Heart Association and CDC recommended guidelines for the incorporation of CRP into its cardiovascular disease risk stratification [9].

Although the relationship between HCV infection and CRP exists in the patient population, studies are limited in the general population. Abaete De los Santos et al (2017) suggested that HCV by itself does not arouse inflammatory reaction in dialysis patients, except in the presence of another ongoing infection or inflammatory condition [18]. Further, another study showed that CRP levels were lower among persons who were HCV positive in most of the patient population studies [19]. To determine whether CRP levels varied by HCV RNA, a study by Tuncer et al (2011) found that CRP levels were higher among HCV-RNA positive group compared to HCV-RNA negative group [20]. Another study also noted that among the HCV-infected women, higher HCV-RNA levels were associated with 9% lower CRP levels [21]. On the contrary, CRP was a prediction factor for disease progression among chronic HCV patients [22].

HCV genotype serves as a prognostic factor that is related to the chronicity of the infection. The most common HCV genotypes in the U.S. population were genotypes 1, 2, and 3 [23]. HCV genotype 1 has been reported to be more aggressive, as it increases the risk for hepatocellular carcinoma, and has a higher tendency to chronicity [23].

As conflicting results were noted in the patient population, an investigation is needed to see the relationship between CRP and HCV infection in the general population. It was hypothesized changes in CRP levels are genotype-dependent in the general population with HCV infection. Therefore, the aims of this study were to: (1) determine prevalence of HCV infection in the general population; (2) compare CRP levels by HCV infection measured by anti-HCV antibody positive and negative status and HCV-RNA positive and negative individuals; and (3) identify the association of CRP in individuals with different HCV genotypes.

## 2. Materials and Methods

### 2.1. Study Population

Data were extracted from the National Health and Nutrition Examination Survey (NHANES) 2009–2010 which is a cross-sectional survey designed to monitor the health and nutritional status of the civilian, noninstitutionalized U.S. population. The NHANES, conducted by the National Center for Health Statistics of CDC, is ongoing health, and nutrition survey which began in the early 1960s. The NHANES uses a stratified multistage probability sampling design which combines interviews and physical examination. A complete description of the methodology is published on the NHANES website [24]. The questionnaire is administered by trained household interviewers using the Computer-Assisted Personal Interviewing (CAPI) system, and participants undergo the medical examination part in the Mobile Examination Clinics (MECs).

### 2.2. Measurements

#### 2.2.1. C-Reactive Protein (CRP)

CRP is a measure of the body’s response to the acute phase of infectious diseases, inflammation from acute and chronic diseases, and environmental exposure to irritants. Blood samples were obtained from participants of NHANES. CRP levels were measured by latex-enhanced nephelometry, which is a reaction between a soluble analyte and its corresponding antigen or antibody bound to polystyrene particles. CRP was quantified through covalently linking of anti-CRP antibodies with a hydrophilic shell and the polystyrene core of CRP particles. The analyses were performed at the laboratory of the University of Washington, Seattle, WA [24]. According to the 2013 American College of Cardiology guidelines and American Heart Association, the median CRP levels, when there is no ongoing acute or chronic infections, chronic conditions, and trauma are about 0.2 mg/dL [25]. In this study, the CRP value is categorized as high (≥0.22 mg/dL) and low (<0.22 mg/dL) based on previous study [26]. 

#### 2.2.2. Hepatitis-C Virus (HCV)

The clinical definition of hepatitis C infection is an acute illness. The laboratory procedures used are described in details on the CDC/NHANES website [24], and the criteria for diagnosis include: (a) antibodies to hepatitis C virus (anti-HCV); (b) a positive HCV recombinant assay (HCV RIBA); and (c) a positive HCV-RNA from the Nucleic Acid Test (NAT). HCV infection can be highly asymptomatic. The serum specimens obtained from participants were processed and transported to the Division of Viral Hepatitis, National Center of HIV/AIDS, Viral Hepatitis, STD, TB Prevention. Serum samples that tested positive for HCV using the VITROS Anti-HCV assay were confirmed with the RIBA confirmatory assay (Chiron RIBA 3.0 Strip Immunoblot Assay). Then HCV positive samples were further tested for HCV-RNA.

The HCV genotype test was performed on samples that tested positive or were indeterminate to HCV-RNA, using the VERSANT ® HCV Genotype 2.0 Assay (LiPA) [24]. For this study, HCV genotypes were classified as: genotype 1 (combining HCV subtype 1a, 1b, and other than 1a, 1b or with different undetermined subtypes), genotype 2 or genotype 3.

#### 2.2.3. Covariates

Age, sex, blood transfusion history, men who have sex with other men (MSM) status and lifestyle behaviors like smoking, alcohol use, injection-drug use were considered as covariates. Age was categorized into two groups: 30 years old or less, as “younger,” and older than 30 years old, as “older.” Participants who had a history of blood transfusion anytime was coded as “1”, and those who have never received blood were coded as “2”. Sex of participants was coded as “1” for females and “2” for males.

Smoking data contained information on participants from ages 12 years and older. During the MEC interview, participants between ages 12 and 19 years used the Audio Computer-Assisted Self-Interviewing (ACASI) system, while participants who were older than 19 years answered the questionnaire on the Computer-Assisted Self-Interviewing (CAPI). The item on the interview was if participants have smoked at least 100 cigarettes in life. Drug use was assessed among participants who were aged 18 years and above. Questions about drug use were asked during the MEC interview at the mobile examination center, for this variable, the item in the questionnaire asked participants about “Ever used a needle to inject illegal drugs.” MSM participants were determined through interviews by asking the question “Have you ever had any kind of sex with a man, including oral or anal?”

#### 2.2.4. Statistical Analyses

Data were analyzed using SAS statistical software (version 9.4 for Windows, SAS Institute Inc., Cary, NC, USA, 2012). Proc survey frequency, means, and regression were used due to the complex survey design of NHANES. Descriptive analyses were performed using proc survey frequency and mean procedure in the weighted sample. Bivariate analyses were performed using proc survey linear regression to observe the association of CRP and HCV status. Then proc survey multivariate regression was applied to see the association between CRP and HCV while adjusting for race, sex, age category, and risk factor variables. Log transformation was applied for CRP variable that was not normally distributed. The weighted percentage and mean values were reported. Two-sided p-values of all tests were considered significant. Collinearity was checked and residual analysis was also performed to check the normality of residuals.

## 3. Results

### 3.1. Sample Characteristics

In 2009–2010 NHANES data, CRP levels were measured in 5611 participants, which made up our sample size. The socio-demographical characteristics of participants are presented in Table 1. Almost 22% of participants were aged 30 years and less, while the remaining 78% (*n* = 4370) were older than 30 years old. The gender distribution was similar, as there were 2885 females (51%), and 2726 males (49%). In this sample, 67% were Whites, 14% were Hispanics, 11% were African Americans and 7% were of other races. 10% of the participants have received blood transfusions in the past, 2% were injection drug users, 4% were MSM, and less than 1% were HIV infected individuals. About 2% of our participants had infected with the hepatitis C virus and 58% of these participants had CRP levels > 0.22 mg/dL.

### 3.2. Bivariate Analyses

#### 3.2.1. Mean CRP Levels among Participants

The mean CRP among participants who were HCV positive was 0.12 ± 0.08 mg/dL, and median CRP levels among this group were 0.12mg/dL. For HCV negative participants, the mean CRP levels were higher, 0.24 ± 0.02 mg/dL and median CRP levels in this group was 0.16 (Table 2).

#### 3.2.2. CRP Levels with Risk Factors

CRP levels were compared with risk factor variables in Table 3. Mean CRP levels differed significantly by race, *p* < 0.001. Mean CRP levels were significantly higher among females compared to males. Mean CRP levels were higher among participants 30 years old and older compared to those who were younger than 30 years. We found higher mean CRP levels among those living with HIV compared to HIV negative persons. We found significantly lower mean CRP levels among MSM compared to those who were non-MSM. For people who had a history of blood transfusion, mean CRP was significantly higher compared to those have not received blood in the past.

### 3.3. CRP levels by HCV-RNA

In the subgroup analysis, out of 93 participants, 71 percent were HCV-RNA positive and 29 percent were HCV-RNA negative. In Table 4, the mean ± SD and selected percentiles of hs-CRP (mg/dL) in the study cohort by HCV-RNA status were presented. The results showed that there were statistically non-significant (*p* = 0.62) differences in the hs-CRP levels between HCV-RNA positive and HCV-RNA negative participants, though median values were higher among HCV-RNA negative individuals, higher values were noted among HCV-RNA positive participants after adjusting for race, sex, age, blood transfusion, and MSM. 

### 3.4. CRP Levels by HCV Genotype

**Figure 1:**
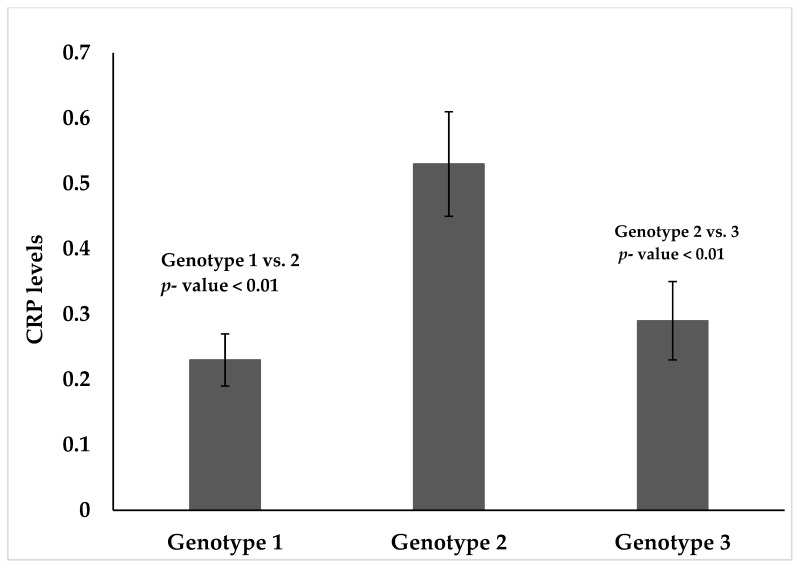
CRP levels by HCV genotypes adjusted for race, sex, and age.

The hepatitis C virus genotype distribution among infected persons in this population was presented in Figure 1. The weighted percentage for genotype 1 was 72.7%, genotype 2 was 9.3% and genotype 3 was 17.3%. Mean CRP levels were significantly higher in people with HCV genotype 2 compared with people with HCV genotype 1, (0.53 ± 0.06 vs. 0.23 ± 0.05, *p* < 0.01, 95% CI: −0.58 to −0.02), and HCV genotype 2 compared with genotype 3 (0.53 ± 0.06, 0.28 ± 0.04, *p* < 0.01, 95% CI: −0.02 to −0.48). There was no statistical significance differences in CRP levels for HCV genotype 3 vs. genotype 1 (0.28 ± 0.04, 0.23 ± 0.05, *p* = 0.42, 95% −0.21 to 0.11) after race, sex, and age had been adjusted for.

## 4. Discussion

This study demonstrated that CRP levels were lower in people with anti-HCV antibodies, although this was statistically non-significant. Lower values of CRP in this group have also been noted in some other studies [18,19]. In acute infection, CRP levels appear early and are expected to increase. Measurement of this biomarker is generally recognized and used in the clinical setting [9,27]. In a prospective study, Mitra et al., (1998) showed a significantly higher level of CRP in children with the acute inflammatory stage of shigellosis, which is a bacterial infection.

In terms of HCV-RNA status, higher values of CRP were noted among subjects with HCV-RNA positive compared to those with HCV-RNA negative status, which is consistent with the study [20], but contradicted another study [21]. But median values of CRP were noted to be higher among HCV-RNA positive individuals. With respect to genotype, CRP levels were significantly higher in people with HCV genotype 2 compared with HCV genotype 1 and 3. CRP is a clinically useful biomarker that indicates the presence of an ongoing acute infection.

Interleukin 6 (IL-6), a pro-inflammatory cytokine, plays an important role of regulating the acute phase response during inflammation and infection. CRP is one of the acute phase proteins that IL-6 stimulates during inflammation [28,29]. In HCV infection, however, the levels of these biomarkers have been seen to decrease significantly [19]. The rationale that has been provided for these observations is associated with the “immune tolerance” mechanism, in which continued replication of the hepatitis C virus in the liver cells results in an alteration in the local immune response in the liver [21,30]. Thus, IL-6 production becomes depressed; this, in turn, would lead to an inhibition in the production of inflammatory proteins, including CRP. This may be a plausible explanation for the decrease in CRP levels seen in HCV infected persons [21,31].

Although confirmatory antibody test may reflect acute HCV status, we compared CRP levels with HCV-RNA status, which may reflect a chronic infection. This further provides evidence of immune tolerance, as seen in similar studies [21]. In the presence of long-standing HCV infection, which is quite common since the infection remains asymptomatic for a long time, the continued replication of the virus in the liver cells may decrease their ability to produce IL-6, and consequently CRP [28,29,31]. This explanation has been said to be contributory, and not sufficient in itself, as experimental and mechanistic studies are needed to test this phenomenon [29].

## 5. Strengths

This study is a population-based national study, a representative sample of the US adult non-institutionalized population (*n* = 5611). The measurement of CRP has been validated in a previous study [9]. HCV antibodies and antigens were measured with validated lab procedures [24].

## 6. Limitations

Because of the unavailability of data on viral load, we could not provide the CRP status with viral load. In addition, this is being a cross-sectional study, the causality of changes in CRP cannot be established solely by HCV status. 

## 7. Conclusions

In conclusion, we found that CRP levels were lower in the presence of HCV infection, although this was statistically non-significant. With respect to HCV-RNA, higher values of CRP were noted, but this was also not statistically significant. When stratified by genotype distribution, mean CRP levels were significantly lower with infection with HCV genotype 1 and genotype 3 compared to HCV genotype 2. We suggest that further studies are needed to confirm these findings. From a public health and clinical perspective, it is important to know the expected mean CRP levels by HCV antibody, HCV-RNA, and genotype distribution.

## Figures and Tables

**Table 1:** Demographic characteristics of participants.

Variables	Categories	Frequency	Weighted Frequency	Weighted Percent
Age (years)				
	< 30	1241	46,010,147	22.42
	≥ 30	4370	159,146,706	77.57
Sex				
	Female	2726	100,219,509	51.14
	Male	2885	104,937,345	48.85
Race				
	Hispanics	1703	29,185,508	14.22
	Whites	2575	138,328,013	67.42
	African American	1013	23,061,139	11.24
	Other races	320	14,582,193	7.10
Received Blood Transfusion				
	Yes	590	19,569,140	9.65
	No	4956	183,052,596	90.34
Injection Drug Use				
	Yes	79	3,113,755	1.85
	No	4287	164,724,273	93.14
MSM				
	Yes	100	3,716,176	4.38
	No	2079	80,966,474	95.61
Smoking				
	Yes	1013	35,834,131	40.26
	No	1459	53,160,492	59.73
HIV				
	Yes	21	641,006	0.39
	No	4021	160,114,320	99.60
Hepatitis C Virus				
	Yes	106	3,322,600	1.61
	No	5505	201,834,254	98.4
C-Reactive Protein				
	≤ 0.22mg/dL	2552	85,411,403	41.63
	> 0.22mg/dL	3059	119,745,451	58.36

**Table 2:** Weighted mean and selected percentile of C-reactive protein (mg/dL by hepatitis C virus (HCV) status): National and Health Nutrition Examination (NHANES) 2009–2010.

Categories	Mean ± SD * (mg/dL)	Selected Percentiles				
		10th	25th	Median	75th	90th
HCV Positive	0.12 ± 0.08	0.01	0.06	0.12	0.28	0.81
HCV Negative	0.24 ± 0.02	0.02	0.06	0.16	0.39	0.88

* *p* = 0.08 and 95% CI for the mean difference (−1.12 to −0.07) after adjusted for race, sex, age, blood transfusion and men who have sex with other men (MSM) status.

**Table 3:** C-reactive protein (CRP) levels by Risk Factor Variables.

Risk Factor Variables	Means ± SD (mg/dL)	*p*-Value
Race		<0.001
Hispanic	0.39 ± 0.03	
African American	0.50 ± 0.04	
White	0.35 ± 0.02	
Other	0.20 ± 0.02	
Sex		<0.001
Male	0.30 ± 0.01	
Female	0.42 ± 0.01	
Age		<0.05
<30	0.32 ± 0.02	
≥30	0.38 ± 0.02	
HIV		0.82
No	0.35 ± 0.02	
Yes	0.36 ± 0.07	
Drug use		0.73
No	0.36 ± 0.02	
Yes	0.38 ± 0.06	
Smoking		0.15
No	0.38 ± 0.02	
Yes	0.43 ±0.03	
MSM		<0.02
No	0.30 ± 0.02	
Yes	0.22 ± 0.04	
Blood Transfusion		<0.02
Received	0.48 ± 0.05	
Did not receive	0.35 ± 0.01	

**Table 4:** Mean and selected percentile of C-reactive protein (mg/dL by HCV-RNA status): NHANES 2009–2010.

Categories	Mean ± SD * (mg/dL)	Selected Percentiles				
		10th	25th	Median	75th	90th
HCV RNA positive	0.56 ± 0.03	0.01	0.05	0.12	0.28	0.61
HCV RNA Negative	0.48 ± 0.05	0.02	0.07	0.19	0.43	1.18

* *p* = 0.62 and 95% CI (−1.37 to 0.86) for the mean difference after adjusting for race, sex, and age, blood transfusion, and MSM.
